# Machine learning-aided risk prediction for metabolic syndrome based on 3 years study

**DOI:** 10.1038/s41598-022-06235-2

**Published:** 2022-02-10

**Authors:** Haizhen Yang, Baoxian Yu, Ping OUYang, Xiaoxi Li, Xiaoying Lai, Guishan Zhang, Han Zhang

**Affiliations:** 1 School of Physics and Telecommunication Engineering, South China Normal University (SCNU), Guangzhou, 510006 China; 2 School of Electronics and Information Engineering, SCNU, Foshan, 528225 China; 3 Guangdong Provincial Engineering Technology Research Center of Cardiovascular Individual Medicine & Big Data, SCNU, Guangzhou, 510006 China; 4 Department of Health Management, Nanfang Hospital, Southern Medical University, Guangzhou, 510515 China; 5 Key Laboratory of Digital Signal and Image Processing of Guangdong Provincial, College of Engineering, Shantou University, Shantou, 515063 China

**Keywords:** Diseases, Endocrinology, Health care, Medical research, Risk factors

## Abstract

Metabolic syndrome (MetS) is a group of physiological states of metabolic disorders, which may increase the risk of diabetes, cardiovascular and other diseases. Therefore, it is of great significance to predict the onset of MetS and the corresponding risk factors. In this study, we investigate the risk prediction for MetS using a data set of 67,730 samples with physical examination records of three consecutive years provided by the Department of Health Management, Nanfang Hospital, Southern Medical University, P.R. China. Specifically, the prediction for MetS takes the numerical features of examination records as well as the differential features by using the examination records over the past two consecutive years, namely, the differential numerical feature (DNF) and the differential state feature (DSF), and the risk factors of the above features w.r.t different ages and genders are statistically analyzed. From numerical results, it is shown that the proposed DSF in addition to the numerical feature of examination records, significantly contributes to the risk prediction of MetS. Additionally, the proposed scheme, by using the proposed features, yields a superior performance to the state-of-the-art MetS prediction model, which provides the potential of effective prescreening the occurrence of MetS.

## Introduction

Metabolic syndrome (MetS) is a series of metabolic disorders of proteins, fats, carbohydrates and other natural substances^1^. It has a high prevalence worldwide and the morbidity is still increasing^2,3^. The aetiology of MetS is complex, and it has been widely recognized that the formation of MetS is related to insulin resistance, obesity, hypertension, and dyslipidemia^4,5^. Besides, it has been pointed out in^6–9^, that MetS may increase the risk of diabetes, cardiovascular diseases (CVDs), chronic kidney diseases and cancers, where the above diseases seriously endanger human’s health due to high mortality^10^. Therefore, it is significant to predict the onset of MetS in advance, which can prevent it from evolving into more serious diseases by early intervention and treatment.

Statistical methods have been widely used to identify the risk factors of MetS in various perspectives. Risk ratio is a commonly used method. Scuteri et al.^11^ used a logistic regression model to derive relative risk (RR) of demographics and MetS components, and obtained that waist circumference (WC), triglyceride (TG), high density lipoprotein cholesterol (HDL-C) are the independent predictors of MetS. Wu^12^ considered the odds ratio (OR) of cardiopulmonary fitness data to the risk of MetS 2 years later in the Taiwan military population. One traditional method for risk prediction is to set risk rules artificially. Taking an example of MetS risk prediction, Zou et al.^13^ set different risk scores for 4 MetS-related risk variables based on hazard ratio (HR) obtained from multiple logistic regression model, and then provided a risk model corresponding to the cumulative risk of these indicators, with the area under the receiver operating characteristic curve (AUC) of 0.690. Another traditional risk prediction method is based on the cut-off value of a single variable. For example, Jowitt et al.^14^ obtained the cut-off point of body mass index (BMI), WC, waist to hip ratio (WHR), waist to height ratio (WHtR) and total body fat (TBF) from previous studies, by which to determine the risk to MetS, and further to predict the occurrence of diabetes and CVDs. These models provided broad perspectives on the risk factors of MetS, but the prediction for the onset is not accurate enough for practical purposes due to the simple binary division of each variable. To address the above issue, Jeong et al.^15^ proposed an areal similarity degree-based model to identify the high-risk group of MetS using a weighted radar chart, where different importance of each variable as well as continuous numerical input was considered.

Machine learning has been regarded as a promising technique due to its powerful learning capability^16,17^. With the help of machine learning, non-invasive indicators without blood drawing can be applied to predict MetS, enabling early diagnosis on MetS even in the areas with poor medical conditions^18,19^. Besides, this technology has enabled the prediction of MetS to be applied to some uncommon fields like metabolic spectrum^20^ and FibroScan ultrasonic elastography equipment^21^. The above works can achieve accurate identification of MetS. Since MetS are often accompanied by various complications^22,23^, it is of significance for potential MetS patients to provide effective risk prediction in advance.

Empowered by machine learning, researches on risk prediction of MetS have been widely concerned in recent years. Farzaneh et al.^24^ predicted the risk of MetS after 7 years by using anthropometric and some commonly used MetS related clinical examination indicators, and concluded that TG, blood pressure (BP) and BMI are the most important risk factors. Lee et al.^25^ constructed a 2-year risk prediction model of MetS and showed the relationship that weight control in different BMI groups to the reduction of MetS predictive index (MPI) 2 years later. In^26^ and^27^, the genetic information was considered, but the results demonstrated that the diet, lifestyle and clinical information still plays a leading role in the risk prediction of MetS. Based on this fact, Lee et al.^28^ combined the “Sasang constitutional (SC) types” features, which involving facial expressions and body posture into account to achieve a long-range prediction of MetS over 14 years. Li et al.^29^ studied the relationship between children’s retinol binding protein 4 (RBP4) and 10-year risk of MetS. Although the above-mentioned models demonstrated that the relationship between MetS and some key clinical variables, such as TG, BP and BMI, are important for the risk prediction of MetS, the impact of the numerical and state changes of such clinical variables on MetS has not been reported yet.

To address the above issues, this paper concerns with a machine learning-aided longitudinal study on risk prediction of MetS by using a total of three consecutive years examination records of 67,730 individuals. To be specific, in addition to the numerical features of examination records, the numerical changes and the normal/abnormal state changes over the past two consecutive years are employed as features for classification for the prediction of MetS in the forthcoming year. To the best of the authors’ knowledge, it is the largest number of samples involved for MetS risk prediction. From numerical results, it is shown that the proposed risk prediction model yields a higher performance in comparison with the state-of-the-art methods. More importantly, we show that the impact of differential state features (DSFs) w.r.t. the clinical variables, i.e., TG, WC, BP and BMI, in addition to the numerical features of examination records, are significant to the risk of MetS, demonstrating that long-term unhealthy lifestyle over 2 years, regardless of age and gender, leads to a high incidence of MetS.

## Results

### Performance of differential features with different classifiers

**Figure 1:**
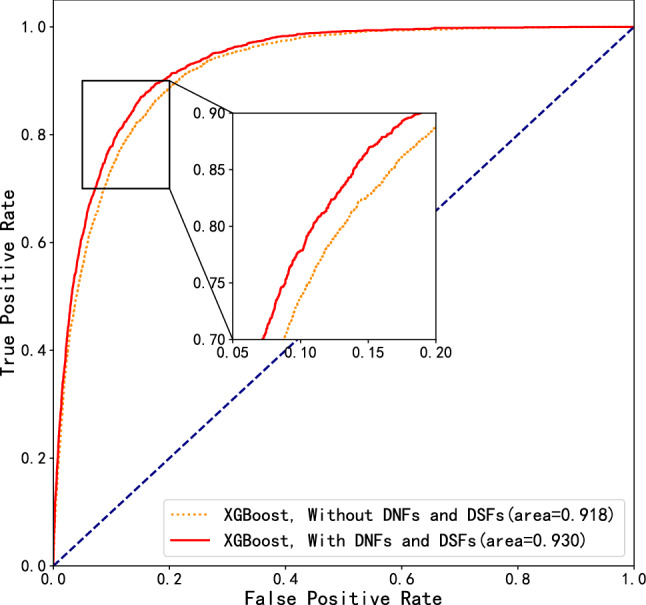
Receiver operating characteristic (ROC) curves of models with/without DNFs and DSFs using XGBoost classifier.

Table 1 shows the performance comparison of MetS prediction models using three different classifiers with and without the proposed differential numerical features (DNFs) and DSFs. For fairness of comparison, all examination indicators of the previous 2 years with and without DNFs and DSFs are considered in experiments. 10-fold cross-validation experiment is carried out, where the metric of AUC is described in mean ± standard deviation (STD), and the best performance in each metric is marked in bold. In addition, we further plot the receiver operating characteristic (ROC) curves of the proposed MetS prediction model with/without the DNFs and DSFs. It can be seen from both Table 1 and Fig. 1 that both the proposed MetS predictive models with and without differential features perform robust with a very small STD value in terms of AUC. The result is reasonable, since the dimension of the dataset employed in this work reaches 67,730 individuals, which is larger than that has been reported by the existing contributions. Furthermore, it can be easily observed that the performance using DNFs and DSFs are superior to that without differential features in terms of all metrics. This result demonstrates that the variations of examination indicators during the consecutive 2 years can be viewed as effective features for predicting MetS in the forthcoming year. In addition, XGBoost performs the best in terms of AUC, Accuracy, Precision, F1-score, Specificity and F2-score, and it yields an AUC and Accuracy of up to 0.930 and 0.849, respectively. It is worth noting that the Precision and F1-score are 0.43 and 0.58 respectively. The result is similar to that of the existing studies^14,19,25,27,30^, and is expected, since the number of positive samples is significantly less than that of negative ones. As a consequence, we select XGBoost as the classifier for the rest experiments unless indicated.

**Table 1:** Results based on three models with and without differential features.

Model	Threshold	AUC	Accuracy	Precision	Recall	F1-score	Specificity	F2-score
**Without DNFs and DSFs**								
XGBoost	0.147	**0**.**918** ± **0**.**003**	**0**.**833**	**0**.**40**	0.85	**0**.**55**	**0**.**83**	**0**.**69**
Stacking	0.116	0.917 ± 0.003	0.812	0.37	**0**.**88**	0.52	0.80	**0**.**69**
Random Forest	0.156	0.908 ± 0.003	0.804	0.36	**0**.**88**	0.51	0.79	0.68
**With DNFs and DSFs**								
XGBoost	0.144	**0**.**930** ± **0**.**002**	**0**.**849**	**0**.**43**	0.87	**0**.**58**	**0**.**85**	**0**.**72**
Stacking	0.125	0.928 ± 0.002	0.837	0.41	**0**.**89**	0.56	0.83	**0**.**72**
Random Forest	0.177	0.916 ± 0.002	0.825	0.39	0.87	0.54	0.82	0.70

The result with the best performance in each metric using different classifiers are marked in bold characters.

### Risk factors of MetS

**Figure 2:**
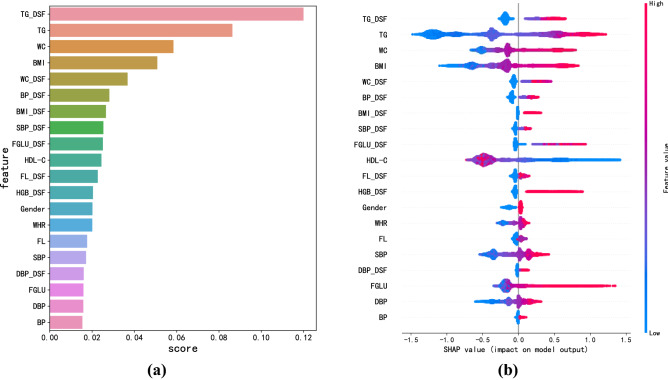
(**a**) Feature importance ranking chart based on XGBoost model (top 20). (**b**) The SHAP analysis of the important features. BMI_DSF, DSF of BMI; BP_DSF, DSF of BP; DBP, diastolic blood pressure; DBP_DSF, DSF of DBP; FGLU, fasting blood glucose; FGLU_DSF, DSF of FGLU; FL_DSF, DSF of FL; HGB_DSF, DSF of hemoglobin (HGB); SBP, systolic blood pressure; SBP_DSF, DSF of SBP; TG_DSF, DSF of TG; WC_DSF, DSF of WC.

As shown in Fig. 2a, we only plot the top 20 important features from all 72 features, since these top 20 features contribute over 90% to the predictive performance of the model.

The 20 features can be divided into two categories: clinical variables and DSFs. The clinical variables include TG, WC, BMI, HDL-C, WHR, FL, SBP, FGLU, DBP, BP, and the DSFs include TG, WC, BP, BMI, SBP, FGLU, FL, HGB, DBP. Notably, the DSFs show strong robustness in the classification results, accounting for 9 out of the top 20 features and 6 out of the top 10 features. However, the DNF shows no obvious contribution to MetS model.

In order to further analyze the contribution of the top 20 features to the prediction of MetS, we provide an explainability analysis using SHAP tool^31^. As shown in Fig. 2b, among all 9 DSFs, the state changes in FGLU and TG contribute the most to the prediction of MetS. By similarity, the examination indicators of FGLU and TG are the top two features with the highest contribution to MetS. In addition to FGLU and TG, both the state changes and the examination indicators of WC and BMI are also important, suggesting that both the conditions whether the values of such indicators exceeding the normal upper limits or the status changes of N2A and A2A over the past 2 years could significantly increase the risk of MetS. It is also noted that the state changes of HGB from N2A and A2A are important features of increasing the risk of MetS, which has not been reported yet.

In view of this, we will further analyze the impact of abnormality in important clinical variables and two differential states (N2A and A2A) of important DSFs on MetS in different gender and age (divided by the world health organization) groups.

### Impact of important clinical variables on MetS risk in different gender and age groups

Firstly, we statistically analyze the risk of MetS in different gender and age groups. As shown in Table 2, the prevalence of MetS for both genders grows with age, and it is higher in male than in female^30^, but the differences are gradually reduced with age growth. For example, for the group aged 18–44, the prevalence ratio of MetS in male is approximately 8 times higher than that of female. For elder age group of more than 60 years old, the prevalence of MetS in male and female are comparable, i.e., 25.41% and 19.07%, respectively. The results are expected, and demonstrate that 20–25% elder people suffers the onset of MetS.

**Table 2:** Prevalence of MetS in the forthcoming year for different gender and age groups.

		Age 18–44	Age 45–59	Age $\ge$ 60
Male	N	23,839	10,540	2975
	Prevalence	13.52%	21.94%	25.41%
Female	N	21,224	6793	1641
	Prevalence	1.67%	6.52%	19.07%

Then, we statistically analyze the contribution of the clinical variables to different gender and age groups, by calculating the odds ratio (OR) of feature’s abnormality to MetS risk in the next year (the largest values of OR in different age groups are bold marked). As can be seen from Table 3 that the main risks of MetS in male aged 18–44 and 45–59 are abnormal TG and BMI. In addition, WC and FL also contribute to the risk of MetS in men under 44 years old. For male group over 60, the risks of MetS in addition to BMI, is mainly due to the abnormality of FL. Besides, the abnormalities of TG and WHR are also relatively important to this group.

**Table 3:** The OR of feature’s abnormality to MetS by age groups in male.

Features	Age 18–44 (95% CI)	Age 45–59 (95% CI)	Age $\ge$ 60 (95% CI)
TG (mmol/L)	**5**.**799** (**5**.**375**–**6**.**257**)	**3**.**498** (**3**.**183**–**3**.**843**)	2.961 (2.426–3.613)
WC (cm)	**4**.**367** (**4**.**012**–**4**.**754**)	2.453 (2.209–2.724)	2.263 (1.848–2.770)
BMI (kg/m^2^)	**5**.**377** (**4**.**802**–**6**.**021**)	**3**.**576** (**3**.**061**–**4**.**177**)	**3**.**488** (**2**.**473**–**4**.**920**)
HDL-C (mmol/L)	2.890 (2.644–3.159)	2.355 (2.067–2.684)	2.478 (1.896–3.237)
WHR (–)	4.021 (3.733–4.331)	2.352 (2.144–2.580)	2.844 (2.440–3.408)
FL (%)	**4**.**259** (**3**.**943**–**4**.**601**)	**3.234 (2.942–3.555)**	**3.055 (2.551–3.660)**
SBP (mmHg)	1.947 (1.796–2.111)	1.656 (1.504–1.824)	1.546 (1.312–1.823)
FGLU (mmol/L)	3.527 (2.840–4.381)	2.762 (2.373–3.216)	2.146 (1.719–2.680)
DBP (mmHg)	3.169 (2.774–3.620)	2.024 (1.778–2.305)	1.686 (1.339–2.122)
BP (%)	2.017 (1.865–2.181)	1.666 (1.517–1.830)	1.578 (1.337–1.861)

The result with the best performance in each metric using different classifiers are marked in bold characters.

Interestingly, it is seen from Table 4 that the most important risk factors of MetS for female aged 18–59 are TG, BMI, FL and FGLU. As age grows, WHR, in comparison with BMI, contribute more significance to the risk of MetS for female aged $\ge$ 45. For elder age group of $\ge$ 60, the most important clinical variables are HDL-C and WHR, respectively. From the aspect of age groups, it is observed that, (1) the impact of clinical variables on younger female (i.e., < 45) is more significant to that on elder ones. (2) The impact of clinical variables on the risk of MetS for female is more significant to that for male of the same age groups.

**Table 4:** The OR of feature’s abnormality to MetS by age groups in female.

Features	Age 18–44 (95% CI)	Age 45–59 (95% CI)	Age $\ge$ 60 (95% CI)
TG (mmol/L)	**13.503** (**10.895**–**16.735**)	**6.633** (**5.460**–**8.058**)	2.337 (1.777–3.074)
WC (cm)	9.177 (7.176–11.735)	5.138 (4.164–6.339)	3.402 (2.557–4.524)
BMI (kg/m^2^)	**13.918** (**10.288**–**18.829**)	**5.854** (**4.387**–**7.810**)	4.060 (2.653–6.213)
HDL-C (mmol/L)	8.257 (6.445–10.579)	4.356 (3.076–6.168)	**6.441** (**3.302**–**12.564**)
WHR (–)	6.100 (4.586–8.115)	**6.515** (**4.510**–**9.410**)	**5.338** (**3.072**–**9.276**)
FL (%)	**15.254** (**11.978**–**19.426**)	**8.100** (**6.513**–**10.073**)	3.537 (2.644–4.731)
SBP (mmHg)	4.618 (3.582–5.955)	2.561 (2.111–3.107)	2.072 (1.615–2.659)
FGLU (mmol/L)	**14.436** (**9.785**–**21.296**)	**5.935** (**4.505**–**7.820**)	2.597 (1.843–3.658)
DBP (mmHg)	7.171 (4.673–11.005)	2.884 (2.118–3.926)	2.223 (1.426–3.467)
BP (%)	4.410 (3.461–5.619)	2.628 (2.172–3.180)	2.154 (1.676–2.767)

The result with the best performance in each metric using different classifiers are marked in bold characters.

The above observations are expected and can be explained as follows. Elder female, in comparison with younger female, generally suffer from more concomitant diseases, of which the influences could potentially neutralize the contribution of single clinical variable on the risk of MetS. By similarity, the prevalence of male suffering from MetS is higher than that of female of the same age groups, and thus, the contribution of clinical variables to male are less obvious than female.

From the results in Tables 2, 3 and 4, it is shown that, the risks of MetS in female with abnormal clinical variables are higher than that in male of the same age groups, but the true prevalence of MetS in female is lower than male group. The potential reason is that, male groups, in comparison with female of the same age groups, generally have irregular diets and unhealthy lifestyle^16^, such as drinking, smoking, etc. Besides, for young and middle-aged female groups, the self-protection mechanism of female’s estrogen^16,32^ is also an important reason for the low prevalence of MetS.

### Impact of important DSFs on MetS risk in different gender and age groups

Next, we statistically analyze the impact that DSFs’ abnormalities have on the MetS of different gender and age groups. The results are shown in Tables 5 and 6, respectively.

Recall the definition of DSF in Eq. (2), the features include N2A (represents specific clinical variable is abnormal in recent 1 year), A2A (represents specific clinical variables are abnormal for past 2 years) and N2N (represents specific clinical variables are normal for past 2 years). For analysis, we evaluate the OR of DSFs’ abnormal states (N2A and A2A) of different gender and age groups by taking N2N state as a control group. For ease of analysis, the two largest values of OR w.r.t. N2A and A2A in different age groups are bold marked, respectively, and the values of OR w.r.t N2A higher than A2A are underlined.

For male aged 18–44, TG and BMI in N2A state have a relative high risk of MetS, and they have the highest risk when in A2A state. In addition, all the features show that compared with abnormality in the only recent 1 year, the risk of people with abnormality in both 2 years was significantly increased. It is still applicable to male over 45 years old. The difference is that with the increase of age, the risk of BMI in A2A state significantly reduced, even less than in the N2A state. And FGLU showed similar characteristics in male over 60 years old. This means that middle-aged and elderly male may have universal abnormal body weight, and the contribution to MetS is relatively stable when there is no significant change in this feature. Similarly, elderly male should also be aware of the significant changes in FGLU. A2A states of TG and HGB hold the highest risks in this age group.

**Table 5:** The OR of the presistent abnormality (A2A) compared to sudden abnormal state (N2A) in male.

Features	Age 18–44		Age 45–59		Age $\ge$ 60	
	N2A	A2A	N2A	A2A	N2A	A2A
TG_DSF	**4**.**661**	**9**.**092**	2.810	**5**.**174**	2.501	**3**.**654**
WC_DSF	3.498	5.827	2.235	3.017	1.954	2.815
BP_DSF	1.807	2.632	1.403	2.065	1.512	1.932
BMI_DSF	**4**.**071**	**6**.**371**	$\underline{\mathbf{3 .\mathbf{802} }}$	$\underline{3.664}$	$\underline{\mathbf{4 .\mathbf{006} }}$	$\underline{3.284}$
SBP_DSF	1.675	2.581	1.484	2.027	1.457	1.930
FGLU_DSF	2.591	5.319	2.592	3.051	$\underline{2.693}$	$\underline{1.996}$
FL_DSF	3.319	6.034	2.507	4.338	**3**.**209**	3.260
HGB_DSF	2.792	4.475	**3**.**399**	**5**.**709**	2.370	**4**.**391**
DBP_DSF	2.348	3.663	1.513	2.195	1.240	1.871

The result with the best performance in each metric using different classifiers are marked in bold characters.

**Table 6:** The OR of the presistent abnormality (A2A) compared to sudden abnormal state (N2A) in female.

Features	Age 18–44		Age 45–59		Age $\ge$ 60	
	N2A	A2A	N2A	A2A	N2A	A2A
TG_DSF	**10**.**037**	**30**.**415**	5.826	**10**.**965**	1.683	3.116
WC_DSF	6.916	17.346	4.870	6.689	**2**.**243**	4.691
BP_DSF	3.585	7.419	2.769	3.258	1.837	3.152
BMI_DSF	**12**.**439**	16.165	$\underline{\mathbf{6 .\mathbf{139} }}$	$\underline{6.000}$	1.028	**6**.**827**
SBP_DSF	3.859	7.728	2.719	3.232	1.605	3.148
FGLU_DSF	9.978	**26**.**366**	4.512	8.325	$\underline{\mathbf{3 .\mathbf{062} }}$	$\underline{2.423}$
FL_DSF	9.287	23.430	**5**.**866**	**11**.**875**	2.119	**4**.**764**
HGB_DSF	3.341	6.003	2.154	3.511	2.007	3.344
DBP_DSF	4.182	7.233	2.682	3.655	$\underline{1.991}$	$\underline{1.887}$

The result with the best performance in each metric using different classifiers are marked in bold characters.

It can be seen from Table 6 that for female aged from 18 to 44, the abnormality of TG, BMI, FGLU and FL lead to a higher risk of MetS in comparison with other clinical variables. When TG, FGLU and FL were abnormal for two consecutive years, the risk of MetS increased significantly. It is also noted that the impact of the abnormal DSFs in terms of TG, FGLU and FL on female aged from 45 to 59 was similar to that of the clinical variables on female aged from 18 to 44. This means that, benefiting from the protection of estrogen, the incidence of abnormal endocrine indicators in female $\le$ 59 is lower than that in male. Meanwhile, when TG and FL are abnormal for two consecutive years, it reflects that the endocrine mechanism disorder of people has exceeded their ability of self-protection by regulating the level of estrogen, leading to a significant increase in the risk of MetS. For female aged over 60, persistent obesity (associated with the abnormalities of both WC and BMI) and abnormal FL were also important risk factors of MetS.

In summary, the results shown in both Tables 5 and 6 demonstrate that, regardless of age and gender, the abnormal clinical variables of two consecutive years lead to higher MetS risk than that of only a single year. Clearly, the results encourage people to carry out necessary measures to avoid abnormal clinical variables for two consecutive years.

Finally, Table 7 shows the comparison between the proposed MetS predictive model and the state-of-the-art studies. It can be seen from Table 7 that, the proposed method, by taking advantages of the differential features of examination indicators over the past consecutive 2 years, yields the highest performance with AUC up to 0.930. Moreover, it is worth noting that the number of samples in dataset analyzed in this work reaches up to 67,730, which is larger than that has been reported yet. Such a large number of dataset can guarantee the robustness to the risk prediction of MetS.

**Table 7:** Comparison between the proposed MetS model and the state-of-the-art contributions.

References	Interval	Data type	Sample size	Method	Performance
^28^	14 years	Physical examination data, SC types	3529	Logistic regression	AUC = 0.817
^29^	10 years	MetS diagnosis indicators, follow-up time, RBP4	352	Logistic regression	AUC = 0.813
^24^	7 years	Physical examination data	2107	Support Vector Machines	AUC = 0.774
^13^	3 years	BMI, DBP, HDL, FPG	4395	Statistic methods	AUC = 0.680
^25^	2 years	Clinical, diet and anthropometric indicators	27,945	XGBoost	AUC = 0.880
**Ours**	**1 years**	**Physical examination data, differential features**	**67,730**	**XGBoost**	**AUC = 0.930**

The result with the best performance in each metric using different classifiers are marked in bold characters.

## Discussion

Studies have shown that MetS is a major cause of diseases such as diabetes and CVDs. Based on a three-consecutive years longitudinal study, this paper studied the risk prediction by taking advantage of the examination records of the current year as well as the differential features of the past two consecutive years.

Based on XGBoost classifier, the impact of 10 clinical variables with the most importance to the risk of MetS is statistically analyzed on different gender and age groups. Specific observations are summarized as follows. Due to the relatively irregular lifestyle, male suffers from a higher prevalence of MetS in comparison with female of different age groups, suggesting that male should pay more attention to the risk of MetS. Thanks to the protective mechanism of estrogen, the ratio of young-aged female with MetS is significantly lower than other age groups. For elder female aged $\ge$ 60, the prevalence of MetS is approximately to that of male group. As regards male group, BMI^21,33^ and FL^30,34,35^ are critical to the risk of MetS for all age groups. In particular, the prevalence of MetS in young-aged group is sensitive to the abnormal of weight (in terms of BMI, WHR, WC and FL), suggesting that male $\le$ 44 years old should pay more attention to control their weight and shape of body. As regards female group, the abnormalities of endocrine clinical variables (in terms of TG, FL and FGLU) are highly related to the prevalence of MetS, especially for young-aged group, i.e., female $\le$ 44 years old. BMI is also of importance to the risk of MetS. In addition, the abnormality of WHR is more and more important to the risk of MetS as age grows, suggesting that middle-aged and older female should pay more attention to the changes of body shape. Owing to the interaction of concomitant disease, the importance of clinical variables abnormality on the risk of MetS is lower in the elderly than in the young and middle-aged groups.

Furthermore, we take the advantages of DSF w.r.t. the abnormal of clinical variables over the past 2 years, aiming to access the relationship between the DSF of specific clinical variables and the risk of MetS prevalence. Statistical results in terms of OR values w.r.t specific DSFs show that the most of the abnormal states over the past 2 years (A2A) lead to higher risk of MetS in comparison with the abnormal states occured only in recent 1 year (N2A). The result behind the observation suggests that any possible intervention should be carried out to prevent the abnormal state of clinical variables over consecutive 2 years. Additionally, it is observed that the abnormality of HGB lasts for consecutive 2 years significantly increases the risk of MetS for male group aged over 45. This result has not been reported yet, and may be explained by the correlation between HGB abnormalities and the occurrence of insulin resistance or MetS in^36,37^.

More importantly, it is noted that, for BMI and FGLU in middle and old-aged groups (i.e., aged $\ge$ 45), the state N2A yields a higher risk of MetS than A2A, suggesting people of such age groups with normal weight and blood glucose should pay special attention to the abnormal state changes of such clinical variables.

In conclusion, with the help of three consecutive years of physical examination records, this paper analyzed the risk of MetS in different age and gender groups by using machine learning algorithms. The statistical results between the onset of MetS and the specific clinical variables (with corresponding state changes over the past consecutive 2 years) could benefit to understand the relationship between the lifestyle and pathogenesis of MetS.

Last but not least, this study has the following two limitations. Firstly, in view of the normal range of each examination indicator, the considered DNFs by taking the advantages of only numerical difference for two consecutive years could not be sufficient without non-uniform mapping w.r.t the specific range. This could be of the potential reason why the contributions of DNFs are trivial to the prediction of MetS. In further study, the non-uniform mapped w.r.t the numerical range of DNFs will be examined. In addition, all samples of dataset in this study are from Guangdong Province, China, and thus, the experimental results may have regional characteristics.

## Methods

### Diagnostic criteria for MetS

According to the Chinese Guidelines for the Prevention and Treatment of Type 2 Diabetes (2017 edition), people with three or more of the following five conditions can be diagnosed as MetS patients: (1) Abdominal obesity: WC $\ge$ 90/85 cm (male/ female). (2) Hyperglycemia: FGLU $\ge$ 6.1 mmol/L or 2-h postprandial blood glucose (PG) $\ge$ 7.8 mmol/L and (or) treatment of previously diagnosed diabetes. (3) Hypertension: BP $\ge$ 130/85 mmHg and (or) treatment of previously diagnosed hypertension. (4) Fasting TG $\ge$ 1.70 mmol/L. (5) Fasting HDL-C < 1.04 mmol/L.

### Dataset

The data of this study is from the Department of Health Management, Nanfang Hospital, Southern Medical University, P.R. China. It contains 546,918 individuals who participated in physical examinations from 2009 to 2019, with a total of 1,039,564 medical records covering several cities in southern China, including Guangzhou, Foshan, Qingyuan, etc. In this data set, 32% of individuals have more than 1 record, 18% of individuals have 3 or more records.

Since part of the indicators were recorded manually according to tons of physical examination reports, inevitably there will be some mistakes. Then we used the upper and lower thresholds, which were set by doctors according to their experience for filtering of the outliers.

After desensitization, integration and cleaning, we obtained the usable structured data (537,283 records for males, and 403,899 records for females). The detailed statistical characteristics are shown in Table 8. There are 32 raw indicators collected in the examination, including anthropometry, blood parameters, other biochemical indicators, medical histories, gender and age.

**Table 8:** Basic statistical characteristics of the raw data set.

Indicators (unit)	Male (N = 537,283)	Female (N = 403,899)	Indicators (unit)	Male (N = 537,283)	Female (N = 403,899)
Age (year)	39.25 ± 13.68	36.84 ± 13.37	ALT (U/L)	26.18 ± 15.12	15.87 ± 9.15
WC (cm)	83.59 ± 9.29	73.02 ± 8.90	AST (U/L)	24.12 ± 8.09	20.12 ± 6.42
FGLU (mmol/L)	5.05 ± 1.22	4.88 ± 0.91	HGB (g/L)	153.24 ± 11.35	130.76 ± 11.59
PG (mmol/L)	7.36 ± 3.08	6.93 ± 2.67	RBC (10^12^/L)	5.20 ± 0.50	4.57 ± 0.43
DBP (mmHg)	75.27 ± 10.91	69.41 ± 10.04	WBC (10^9^/L)	6.80 ± 1.69	6.26 ± 1.58
SBP (mmHg)	124.00 ± 15.34	114.62 ± 16.04	PLT (10^9^/L)	239.81 ± 54.05	261.42 ± 60.44
BP (ratio)	15.73%	7.79%	CR (μmol/L)	79.25 ± 16.38	54.49 ± 13.73
TG (mmol/L)	1.77 ± 1.59	1.13 ± 0.85	DM_H (ratio)	1.27%	0.60%
HDL-C (mmol/L)	1.27 ± 0.32	1.54 ± 0.37	HTN_H (ratio)	3.74%	1.96%
Hip (cm)	94.38 ± 6.40	90.04 ± 6.49	SMK_H (ratio)	–	–
WHR (–)	1.04 ± 0.20	1.02 ± 0.13	FL (ratio)	32.86%	12.62%
HBA1c (%)	5.87 ± 0.80	5.74 ± 0.69	TN (ratio)	7.92%	13.79%
BMI (kg/m^2^)	24.12 ± 3.36	21.96 ± 3.19	HM (ratio)	–	10.20%
TC (mmol/L)	5.23 ± 1.02	5.03 ± 1.00	MGH (ratio)	–	78.09%
LDL-C (mmol/L)	3.19 ± 0.78	2.89 ± 0.77	UALB (ratio)	2.47%	1.08%
UA (μmol/L)	414.36 ± 88.06	298.97 ± 70.83	MS_result (ratio)	11.48%	2.88%

Continuous indicator is expressed as mean ± standard deviation, discrete indicator is expressed as a percentage (%). MS_result is the numerical result of MetS, the value is 0 or 1, 0 represents no disease, 1 represents disease. – means less than 0.1% of the data is available due to missing or gender specific examinations.

ALT, alanine aminotransferase; AST, aspartate aminotransferase; CR, creatinine; DM_H, history of diabetes mellitus; HBA1c, hemoglobin a1c; HM, hysteromyoma; HTN_H, history of hypertension; LDL-C, low-density lipoprotein cholesterol; MGH, mammary gland hyperplasia; N, numbers; PLT, platelets; RBC, red blood cell count; SMK_H, history of smoking; TC, total cholesterol; TN, thyroid nodules; UA, uric acid; UALB, urine albumin; WBC, white blood cell count.

The study was conducted under the approval of the Academic Committee of South China Normal University (Approval No.: SCNU-PHY-2020-063). All methods we used in the study were adherence to relevant ethical guidelines and regulations (Declaration of Helsinki). All subjects signed an informed consent form before inclusion in the present study.

### Longitudinal MetS risk prediction model

**Figure 3:**
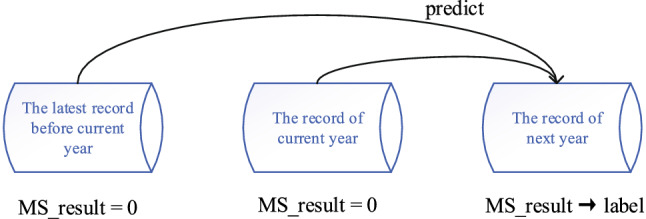
Schematic diagram of the risk prediction model for MetS within the next 1 year.

The risk prediction model for MetS is shown in Fig. 3 (MS_result is the status whether suffering from MetS or not. MS_result = 0 and 1 represent the status with MetS and without MetS, respectively). Unlike the conventional methods, we take both indicators of the current year and the latest one before the current year into consideration in order to obtain features of physical change in time dimension. The prediction can be regarded as a supervised classification, where the status suffering from MetS in the next year is labeled as “1”, and records of the current year and differential features extracted from the past two records as the model input. Thus, a sample contains three records in the model.

Since the risk prediction of MetS represents the process suffering from MetS from a healthy state, the first two records in all three records should be healthy state. Considering the time difference of taking physical examination (usually in the first or third quarter in a year in CHINA), we set the maximum time interval between the first two records and the third one to 540 days.

After the above processing, 67,730 usable samples were obtained, in which the samples with/without MetS are 7971 and 59,759, respectively. For all samples, male and female account for 56% and 44% respectively.

### Feature extraction

Features play a significant role for task classification. In this section, two kinds of differential features in time are proposed, characterizing the deviation of the value and state transition of indicators, respectively.

#### Differential numerical feature (DNF)

The differential numerical feature can be characterized as

1 $$\begin{aligned} I\_{DNF} = \Delta _I= I_0 - I_{-1} \end{aligned}$$

where $I_0$ and $I_{-1}$ denote the values of specific indicator *I* of current year and that of the latest record before current year, respectively.

As a consequence, $I\_{DNF}$ can describe the absolute numerical difference of indicators over years, including the increment, decrement, invariableness, and missing value. This kind of feature is extracted from the indicators with a numerical number, and thus 21 features are extracted.

#### Differential state feature (DSF)

DSF describes the state change process of indicator *I* over the past two examination records, and it can be characterized as

2 $$\begin{aligned} I\_{DSF} = S(I_{-1})\rightarrow S(I_0) \end{aligned}$$

where $S(I_{-1})$ and $S(I_0)$ represent the state of indicator *I* in the latest record before the current year and the current year, respectively, and its values are normal, abnormal or null. We set the upper limit of the clinical reference range of indicators except for HDL-C as the threshold, and beyond the threshold as “abnormal” state, since the increase in the values of indicators is associated with the risk of MetS. Among them, we set threshold of BMI as 28 kg/m^2^. The “abnormal” state of HDL-C is defined as the value lower than its clinical range, since such indicator is protective to MetS.

The status of $I\_{DSF}$ can be normal-to-normal (N2N, represents indicators are normal for past 2 years), normal-to-abnormal (N2A, represents specific indicator is abnormal in recent 1 year), abnormal-to-normal (A2N, represents the indicator changes from abnormal to normal), abnormal-to-abnormal (A2A, represents specific indicators are abnormal for past 2 years) and missing value (specific indicator is empty in either record of the past 2 years). There are 26 DSFs in this paper except for gender, age, hip and three medical histories.

### Dealing with missing value and normalization

The regular physical examination generally involves a fixed part of the items, so the presence of missing values is common in the records, which bring challenges to MetS prediction. In this study, we propose to fill the missing values of indicators based on the following criteria in terms of missing rate, data type and distribution.

- If the amount of missing value is relatively large (70% or more of the data is missing), delete the features directly (in this case, the indicators HBA1c, PG and SMK_H are removed from the dataset.).
- For features with numerical type, fill the missing indicators with the mean values when the values of such group of indicators follow normal distribution (features including BMI, CR, DBP, FGLU, HGB, Hip, LDL-C, PLT, RBC, SBP, TC, UA, WBC, and WC are filled accordingly.). If the values of such indicators follow skewed distribution, use the median to fill in the missing one (Age, ALT, AST, HDL-C, TG).
- For non-numeric data, retain its missing value status and fill in a fixed value (for example, DM_H, HYT_H, FL, TN, HM, MGH, UALB, the DSFs).

For features deleted due to the high missing rate, the corresponding DNF and DSF are also deleted. After the above processing, there are 72 features in total, including 29 raw features, 19 DNFs and 24 DSFs.

**Figure 4:**
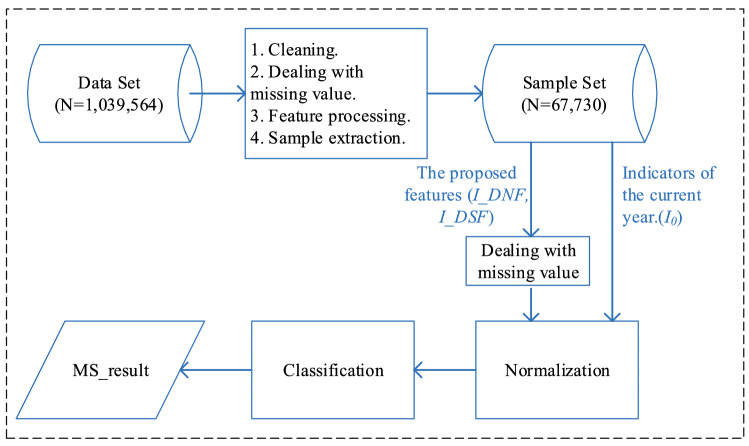
Framework of our MetS risk predictive model.

Finally, we use the standard deviation normalization for features to normalize the contributions of different features to the model. Figure 4 shows the framework of predictive model for MetS based on machine learning techniques.

### Experimental setup

In the experiments, the training set and test set are divided randomly by a ratio of 7 to 3. In order to validate the generalization ability of the model, the age and gender of the samples in the test set and the training set are of the same level.

We use three commonly used decision tree-based ensemble classification algorithms, namely, Random Forest (criterion = ‘entropy’, max_depth = 8, max_features = ‘sqrt’, n_estimators = 500), XGBoost (max_depth = 4, n_estimators = 500, learning_rate = 0.03, colsample_bytree = 0.5) and Stacking (combination of the above two algorithms), to perform the prediction of MetS. Without loss of generality, a threshold of probability should be set for the final decision. In the experiments, the maximum Youden index criteria is employed to determine the optimal threshold.

For measurement, we assess the performance of the proposed MetS prediction model by employing Accuracy, Precision, Recall (Sensitivity), Specificity, F1-score, F2-score (it favors Recall over Precision), which are given as

3 $$\begin{aligned} Accuracy &= \frac{TP+TN}{TP+FP+FN+TN} \end{aligned}$$

4 $$\begin{aligned} Precision & = \frac{TP}{TP+FP} \end{aligned}$$

5 $$\begin{aligned} Recall & = \frac{TP}{TP+FN} \end{aligned}$$

6 $$\begin{aligned} Specificity &= \frac{TN}{TN+FP} \end{aligned}$$

7 $$\begin{aligned} F1{\text{-}}score & = 2 \times \frac{Precision \times Recall}{Precision + Recall} \end{aligned}$$

8 $$\begin{aligned} F2{\text{-}}score & = 5 \times \frac{Precision \times Recall}{4 \times Precision + Recall} \end{aligned}$$

where TP (true positive), TN (true negative), FP (false positive) and FN (false negative) are the values in confusion matrix.

Besides, AUC is used to evaluate the performance of risk prediction. The value of AUC ranges from 0 to 1, and $AUC = 1$ denotes perfect classification.
